# Cellulose fibres, nanofibrils and microfibrils: The morphological sequence of MFC components from a plant physiology and fibre technology point of view

**DOI:** 10.1186/1556-276X-6-417

**Published:** 2011-06-13

**Authors:** Gary Chinga-Carrasco

**Affiliations:** 1 Paper and Fibre Research Institute (PFI AS), Høgskolerringen 6b, 7491 Trondheim, Norway

## Abstract

During the last decade, major efforts have been made to develop adequate and commercially viable processes for disintegrating cellulose fibres into their structural components. Homogenisation of cellulose fibres has been one of the principal applied procedures. Homogenisation has produced materials which may be inhomogeneous, containing fibres, fibres fragments, fibrillar fines and nanofibrils. The material has been denominated microfibrillated cellulose (MFC). In addition, terms relating to the nano-scale have been given to the MFC material. Several modern and high-tech nano-applications have been envisaged for MFC. However, is MFC a nano-structure? It is concluded that MFC materials may be composed of (1) nanofibrils, (2) fibrillar fines, (3) fibre fragments and (4) fibres. This implies that MFC is not necessarily synonymous with nanofibrils, microfibrils or any other cellulose nano-structure. However, properly produced MFC materials contain nano-structures as a main component, i.e. nanofibrils.

## Review

### Introduction

Wood pulp fibres are presently a major area of research for several end-use applications. Fibres can be utilised as reinforcement in bio-degradable composites and as a source of raw materials for bio-energy and biochemical production. Wood pulp fibres have been applied as the raw material for the production of a fibrillated material, which was introduced and defined as microfibrillated cellulose (MFC) by Turbak et al. [1] and Herrick et al. [2]. Several modern and high-tech nano-applications have been envisaged for MFC [1]. Although cellulose fibres have constituted the main source for MFC production, the utilisation of other pulp fibres, agricultural crops and by-products have also been explored [3-5]. With the years, various subjective definitions have been given to the fibrillated materials, e.g. nanofibrillated cellulose, nanofibres, nanofibrils, microfibrils and nanocellulose [6-10].

The German philosopher Immanuel Kant (1724 to 1804) wrote: "Things which we see are not by themselves what we see... It remains completely unknown to us what the objects may be by themselves and apart from the receptivity of our senses. We know nothing but our manner of perceiving them...". Perception is thus a key word with respect to how we subjectively interpret structures. This is clearly exemplified in the relatively large number of terms that have been applied to roughly the same material, and emphasises the necessity of objectively clarifying and standardising the terminology applied within cellulose nanotechnology research. All the given terms relate to structures with nano-dimensions. However, is MFC a nano-structure? The purpose of this review is thus to shed more light on (1) the morphology of MFC structures, (2) the relationship between biological components of fibre wall structures and engineered cellulose-based nano-materials and (3) the terms associated with the MFC denomination. This review will not include other forms of cellulose materials, such as whiskers or bacterial cellulose, which may also be referred to as nanocellulose. For interested readers, see Klemm et al. [5].

### The structure of wood pulp fibres

**Figure 1:**
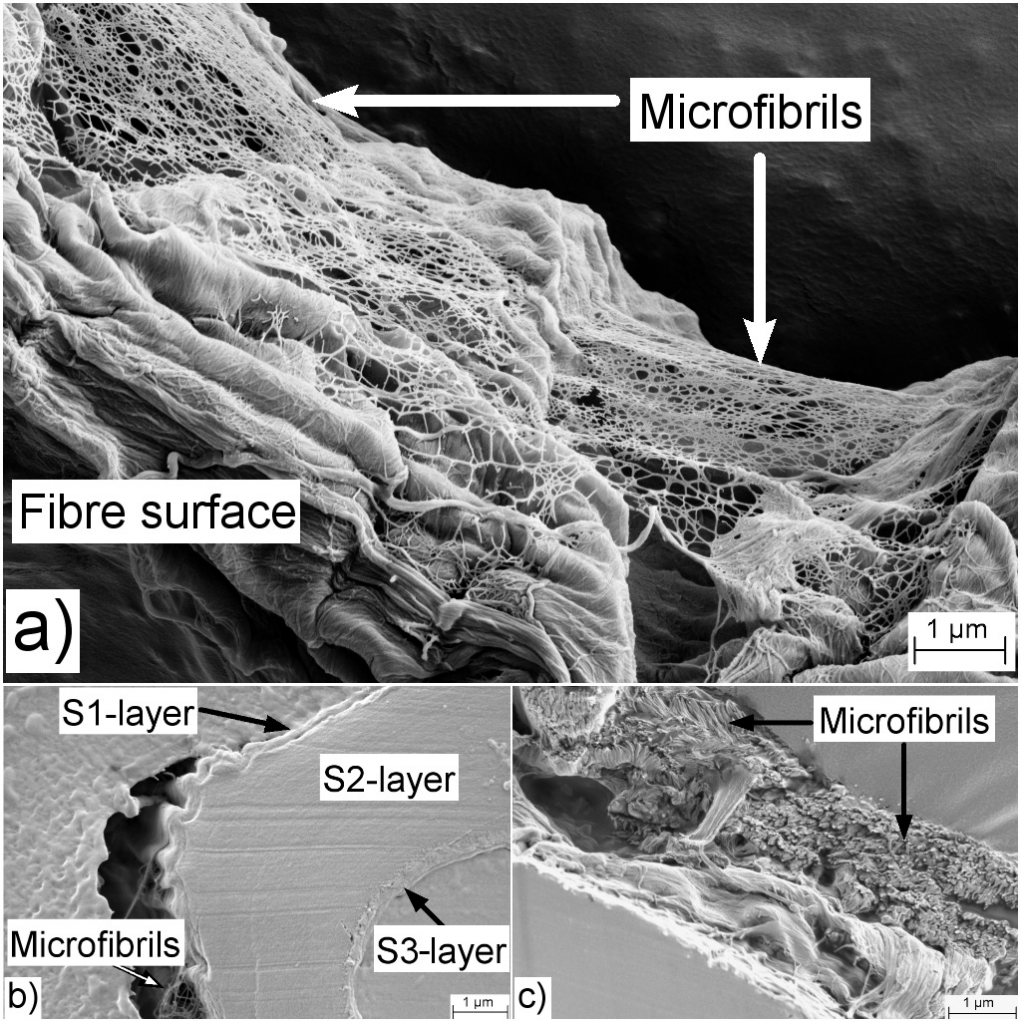
**Structure of wood pulp fibres**. (**a**) Note the network of microfibrils covering the outer wall layer. (**b**) Microtomed cross section showing the S1, S2 and S3 layers. (**c**) Cross-sectional fracture area, showing the microfibrils in the S2 layer. Reproduced and modified from Chinga-Carrasco [11].

The wood pulp fibres have multiscale characteristics [11]. Roughly, typical lengths of fibres are 1 to 3 mm and typical widths are 10 to 50 μm. The fibre wall thickness is roughly between 1 and 5 μm (Figure 1). The fibre wall is composed of defined layers (Figure 1b), including the primary wall (P) and several secondary wall layers (S1, S2 and S3). Each of these layers is characterised by a specific arrangement of fibrils as has been detailed described for more than 40 years ago [12].

Chemical pulp fibres are produced through chemical pulping where lignin and hemicellulose are extracted. Chemical pulp fibres have a surface, which is characterised by a particular pattern created by wrinkles and microfibrils in the outer layers of the fibre wall structure (Figure 1a). The surface structure of chemical pulp fibres corresponds mainly to the primary and S1 layers of the fibre wall, which are preserved during chemical pulping. Contrary to the outer layers of the fibre wall (primary and S1 layers), the S2 layer is characterised by having a structure of microfibrils organised in a helical manner [12].

**Figure 2:**
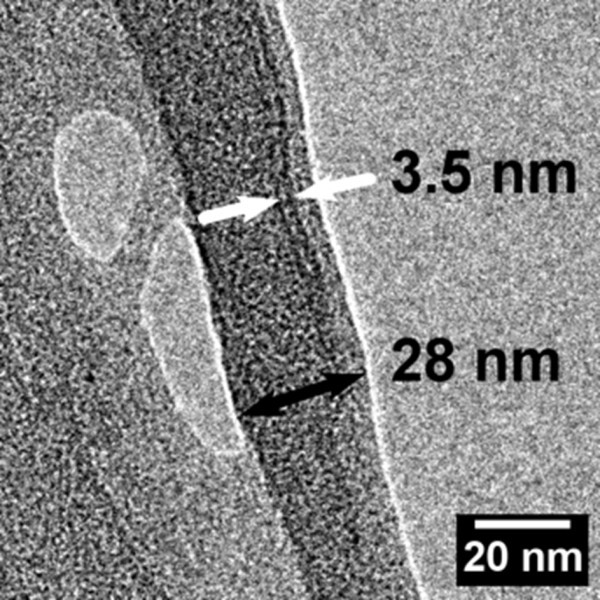
**Microfibril of *Pinus radiata***. Image acquired with TEM. The black arrow indicates the boundaries of a microfibril, which is approximately 28 nm in diameter. The white arrows indicate a single elementary fibril, which is 3.5 nm in diameter. See also Chinga-Carrasco et al. [16].

According to Meier [13], the cellulosic components of a wood fibre wall structure are the cellulose molecule, the elementary fibril, the microfibril, the macrofibril and the lamellar membrane. In the work of Maier [13], the term "elementary fibril" was reported to have a diameter of 3.5 nm and was applied following the terminology of Frey-Wyssling [14]. Heyn [12] stated that elementary fibrils are universal structural units of natural cellulose, as the same biological structure had been encountered in cotton, ramie, jute and wood fibres. Blackwell and Kolpak [15] reported also the occurrence of elementary fibrils with diameters of approximately 3.5 nm in cotton and bacterial cellulose, thus giving supportive evidence about the basic fibrillar unit in cellulose microfibrils, see also [16]. According to Meier [13], microfibrils are agglomerates of elementary fibrils and always have diameters which are multiples of 3.5 nm (Figures 1c and 2). The bundling of elementary fibrils into microfibrils is caused by purely physically conditioned coalescence as a mechanism of reducing the free energy of the surfaces [17]. The maximum diameter of a microfibril was proposed to be 35 nm [13]. Clearly, there has been a debate during the 1950 to 1960s about the terminology applied for describing the elementary components of a plant cell wall. Ohad and Danon [18] applied the microfibril term to the basic plant cell wall structures having a diameter of 3.5 nm, i.e. the elementary fibrils [12,14,19,20]. The microfibril structures reported by Frey-Wyssling [14] were defined as "composite fibres" by Ohad and Danon [18].

Elementary fibrils are generated in complex biological processes, involving cellulose synthase complexes in the plasma membrane, exocytosis of cell wall polymers and cortical microtubules [21]. It seems to exist sufficient evidences that elementary fibrils in vascular plant cell walls are composed of 36 β-1,4-glucan chains, synthesised by the cellulose synthase proteins in the plasma membrane (rosette complexes) [22,23].

### Microfibrillated cellulose

Since the introduction of the transmission electron microscope, it seems that researchers have attempted to disintegrate cellulose fibres into single microfibrils/elementary fibrils for ultrastructural studies. Already in the 1950s, ultrasonic, hydrolysis and oxidation treatments were applied for disintegrating cellulose structures [14,17,24]. In addition, Ross Colvin and Sowden [25] reported a homogenization process based on beating for opening the structure of cellulose fibres and thus exposing the microfibril structures for transmission electron microscopy (TEM) analysis.

The disintegration of cellulose fibres into their structural components (microfibrils) has also found industrial interest. As mentioned above, in 1983, Turbak et al. [1] introduced a homogenisation procedure for fibrillating cellulose fibres with commercial purposes. The MFC terminology, which was originally applied to the fibrillated material, was probably related to the predominant structures encountered in fibre wall structures, i.e. microfibrils [9].

**Figure 3:**
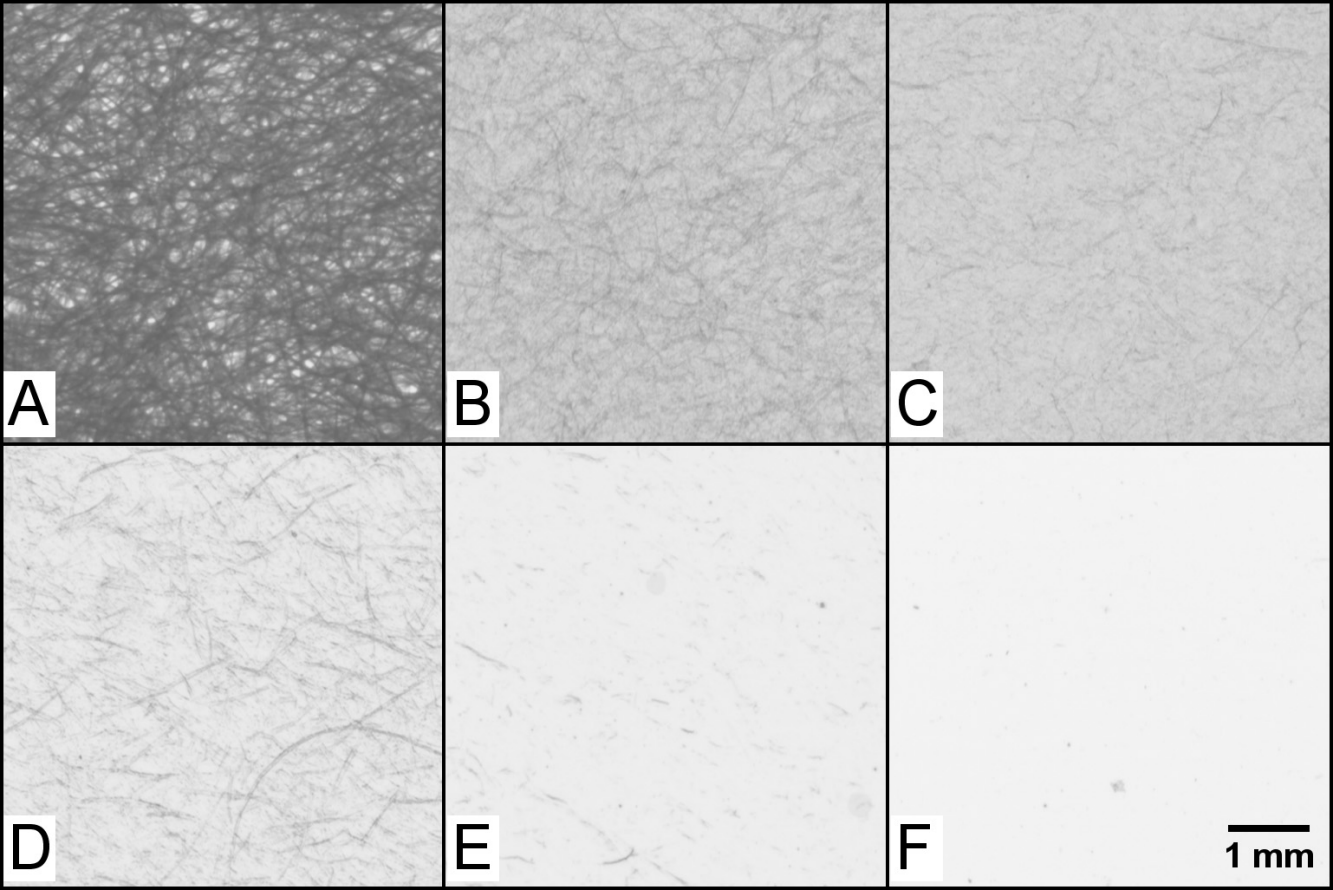
**Films made of cellulose materials with a grammage of 20 g/m^2^**. (**A**) Control film made of 100% *P. radiata *pulp fibres. (**B**) Film made of MFC, homogenised with three passes and 1,000 bar pressure. (**C**) Film made of MFC, homogenised with five passes and 1,000 bar pressure. (**D**) Film made of MFC produced with TEMPO-pre-treated fibres, three passes and 200 bar pressure. (**E**) Film made of MFC produced with TEMPO-pre-treated fibres, three passes and 600 bar pressure. (**D**) Film made of MFC produced with TEMPO-pre-treated fibres, five passes and 1,000 bar pressure. Dark threadlike structures indicate poorly fibrillated fibres or fibre fragments. The lighter the local areas, the higher the transparency levels. For details, see Syverud et al. [35].

**Figure 4:**
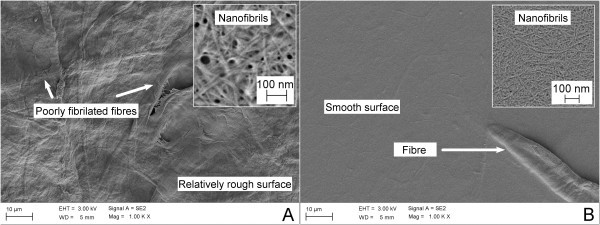
**Surfaces of films (20 g/m^2^) made of microfibrillated cellulose**. (**A**) MFC obtained by mechanical homogenisation. The image corresponds to the film shown in Figure 3C. (**B**) MFC obtained with TEMPO-mediated oxidation as pre-treatment and mechanical homogenisation. The image corresponds to the film shown in Figure 3F. The insets in (A) and (B) represent the surface structure visualised at 50,000× magnification from areas without a metallic coating. Both MFC materials have been collected after passing five times through the homogeniser, at 1,000 bar. For details, see Chinga-Carrasco et al. [16].

**Figure 5:**
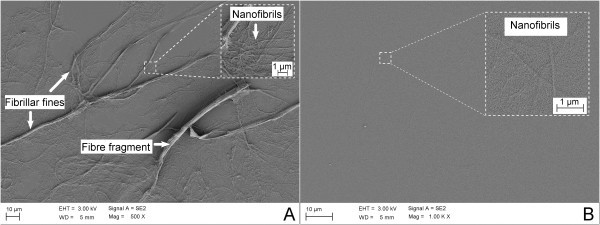
**Microfibrillated cellulose suspensions dried on glass slides**. (**A**) MFC obtained by mechanical homogenisation. Note the relatively large structures remaining after a homogenisation process. The inset shows structures composed mainly of nanofibrils. (**B**) MFC obtained with TEMPO-mediated oxidation as pre-treatment and mechanical homogenisation. The inset shows the nanofibrils having relatively homogeneous sizes. Both MFC materials (A and B) have been collected after passing five times through the homogeniser, at 1,000 bar.

Although microfibrils seem to be the main component of MFC, several studies have shown that fibrillation produces a material which may be inhomogeneous [2,16,26,27], containing, e.g. fibres, fibre fragments, fines and fibrils (Figures 3, 4 and 5). As exemplified in Figure 3, the fraction of each component depends on (1) the treatment applied to the fibres before homogenization, (2) the number of passes through the homogenizer and (3) the pressure applied during homogenization. The more severe the homogenisation, the more fibrillated is the material. Higher degree of fibrillation can be indicated by an increase in the transparencies of the MFC materials due to the generation of optically inactive fibrils. Such fibrils form dense and compact structures, with low light scattering potential.

Having fibrillated materials with different degree of homogenisation and composed of a variety of structures emphasises the necessity of clarifying the different components encountered in MFC. Table 1 gives a rough classification of MFC components, including classical terminology that has been applied in plant physiology for decades and terminology related to fibre technology.

**Table 1:** Components of microfibrillated cellulose

Diameter (μm)	Biological structures	Technological terms
10 to 50	Tracheid	Cellulose fibre
<1	Macrofibrils [13,17,28]	Fibrillar fines, fibrils [29,30]
<0.1		Nanofibril, nanofibres [6,8,16,34]
<0.035	Microfibril [13,15]	
0.0035	Elementary fibril [12-15]	

Terms and sizes according to terminology and morphology reported in the literature.

The fibril term has been applied for defining structures with a dimension less than 1 μm, although not consequently. Structures with diameters of <1.0 μm have also been observed in the fibre wall structure of pulp fibres. Such structures have been denominated macrofibrils, and diameters of approximately 0.66 μm have been reported [28]. However, according to Meier [13], macrofibrils do not have definite dimensions. A fibril may also be considered an engineered structure as it is produced during mechanical fibrillation. There seems to be no concrete border line between fibrils and fibrillar fines (Figure 5A). Fibrillar fines may also be created through refining or beating, from mechanical and chemical pulp fibres, respectively [29]. Subramanian et al. [30] considered fibrillar fines, microfines and microfibrillar cellulose in the same category, i.e. particles that pass a 75-μm diameter round hole or a 200-mesh screen of a fibre length classifier. Such a definition indicates that MFC may also be considered as fines, as exemplified in Figure 5A. Both materials are composed of relatively small and fibrillated components. However, according to Turbak et al. [1], no amount of conventional beating yields the microfibrillation obtained with an optimally homogenised product.

The microfibrillation mentioned by Turbak et al. [1] does not seem to refer to the creation of micrometre-sized particles but to the fibrillation of fibres into individualised microfibrils with diameters less than 100 nm [1]. In this context, it is appropriate to introduce in this review a scale that has been widely emphasised during the last years within modern technology, i.e. "nano". It seems to be widely accepted that a nano-scale refers to sizes between 0.001 and 0.1 μm (1 to 100 nm). This implies that the nanofibril term refers to fibrils with diameters less than 100 nm. Based on this definition, it seems obvious that microfibrils can be considered nanofibrils, which also are composed of crystalline and amorphous regions. However, the difference between microfibrils and nanofibrils is that the former is a well-defined biological structure found in plant cell walls, whereas the latter can be considered a technological term introduced to describe secondary and engineered structures with diameters less than 100 nm.

As mentioned above, conventional MFC production yields materials with inhomogeneous sizes (Figures 3B,C, 4A and 5A). However, the fibrillation can be facilitated by, e.g. pre-treating the cellulose fibres enzymatically [31] or chemically [32,33]. Pre-treatments have thus facilitated the production of homogeneous fibril qualities, with fibril diameters less than 100 nm (Figure 3E,F). In addition, some authors have reported a filtration procedure to remove poorly fibrillated fibres, thus maintaining mostly the fraction of homogeneous nanofibrils [34].

In general terms, the production of homogeneous fibril qualities may require major costs, including costs related to pre-treatments and to energy consumption during production. The less energy that is utilised, the less is the fibrillation of cellulose fibres and the less the amount of produced nanofibrils [35]. Considering that conventional fibrillation (e.g. homogenisation without pre-treatment) produces a material that is inhomogeneous and may contain a major fraction of poorly fibrillated fibres and fines, can we state that MFC is a nano-structure? MFC *per se *is not necessarily a nano-material, but contains nano-structures, i.e. the nanofibrils (Figures 4 and 5). To define MFC as a nano-structure, it is necessary to give substantial evidence with respect to (1) the fraction of fibrillated fibres, (2) the fraction of nanofibrils and (3) the morphology of the nanofibrils in an MFC material. Provided that a given MFC is composed of an appropriate fraction of individualised nanofibrils, the MFC will have a major influence on the rheological, optical, mechanical and barrier properties of the corresponding materials.

Commonly, morphological evidences are given by microscopy and subjective evaluations. Researchers may focus on the visualisation of nano-structures, applying equipment designed for nano-assessment, e.g. FESEM, AFM and TEM. However, such equipment may limit the field of view considerably, which also introduces a subjective pre-selection of small areas containing nano-structures. Proper characterisation requires the quantification of the fibrillated material at several scales. This can include methods for assessing large areas, with a suitable resolution. One important aspect is not only the quantification of nanofibril morphology but also the quantification of fibres that are poorly fibrillated (see, e.g. Figure 3). Methods for assessing relatively large areas and structures at the micrometre scale are thus most valuable for complementing specialised devices for nano-characterisation.

## Conclusions

It has been considered most important to propose an appropriate morphological sequence and definitions of MFC components. Microfibrils are important fibre wall components, i.e. biological nano-structures. However, due to the classical suffix "micro", microfibrils may be wrongly associated with micrometre-sized fibrils, which may be 1,000 times larger (>1 μm). According to evidences given in the literature and personal experience with characterisation of a variety of MFC qualities, it appears that MFC materials may be composed of (1) nanofibrils, (2) fibrillar fines, (3) fibre fragments and (4) fibres. This implies that MFC is not necessarily synonymous with microfibrils, nanofibrils or any other cellulose nano-structure. However, properly produced MFC materials contain nano-structures as a main component, i.e. nanofibrils.

## Abbreviations

MFC: microfibrillated cellulose; TEM: transmission electron microscopy; FESEM: field emission scanning electron microscopy; AFM: atomic force microscopy.

## Competing interests

The author declares that he has no competing interests.
